# MicroRNA-434-3p regulates age-related apoptosis through eIF5A1 in the skeletal muscle

**DOI:** 10.18632/aging.101207

**Published:** 2017-03-22

**Authors:** Patricia S. Pardo, Ameena Hajira, Aladin M. Boriek, Junaith S. Mohamed

**Affiliations:** ^1^ Division of Pulmonary and Critical Care Section, Department of Medicine, Baylor College of Medicine, Houston, Texas 77030, USA; ^2^ Division of Exercise Physiology, Department of Human Performance, West Virginia University School of Medicine, Morgantown, West Virginia 26506, USA; ^3^ Center for Basic and Translational Stroke Research, West Virginia University School of Medicine, Morgantown, West Virginia 26506, USA

**Keywords:** ging, sarcopenia, mitochondria, skeletal muscle

## Abstract

Increased activation of catabolic pathways, including apoptosis causes sarcopenia. However, the precise molecular mechanism that initiates apoptosis during aging is not well understood. Here, we report that aging alters miRNA expression profile in mouse skeletal muscle as evidenced by miRNA microarray and real-time PCR. We identified miR-434-3p as a highly downregulated miRNA in the skeletal muscle of aging mice. Myocytes transfected with miR-434-3p mimic prevents apoptosis induced by various apoptotic stimuli, and co-transfection of miR-434-3p antagomir abolishes the inhibitory role of miR-434-3p. We found that miR-434-3p inhibits apoptosis by targeting the eukaryotic translation initiation factor 5A1 (eIF5A1). Overexpression of miR-434-3p in myocytes reduces the loss of mitochondrial transmembrane potential, and activation of caspases-3, −8 and −9 by suppressing eIF5A1 in response to various apoptotic stimuli whereas inhibition of miR-434-3p reversed this scenario. Skeletal muscles from aging mice exhibit low levels of miR-434-3p and high levels of eIF5A1, suggesting a possible role for miR-434-3p in the initiation of apoptosis in aging muscle. Overall, our data identified for the first time that miR-434-3p is an anti-apoptotic miRNA that may be therapeutically useful for treating muscle atrophy in various pathophysiological conditions, including sarcopenia.

## INTRODUCTION

Sarcopenia is an advanced age-related loss of skeletal muscle mass as well as loss of its function, which limits the independence living and quality of life, thereby it underlies morbidity and mortality in elderly individuals [1, 2]. Sarcopenia also reduces the amount of metabolically active tissue; thus, it increases the risk for metabolic diseases [3]. Initiation of sarcopenia involves complex processes that are controlled by both extrinsic and intrinsic factors [3, 4], many of which converge on a decline in the ability of muscle stem cells (satellite cells) to replace unhealthy muscle fibers during aging [5-7]. Although the mechanism that causes sarcopenia are largely unknown, a progressive decline in anabolism mostly due to a reduction in protein synthesis with an increase of catabolism mainly due to an enhanced activation of pathways like apoptosis, initiate sarcopenia [2]. Apoptosis is essential for organ development, tissue homeostasis, and the elimination of defective cells in multi-cellular organisms; however, accelerated apoptosis in skeletal muscle is a potential mechanism of sarcopenia [8-13]. Different apoptotic stimuli such as oxidative stress, calcium, and TNF-α, may be seen as initiators of the apoptotic signaling in skeletal muscle during aging [8, 9, 14].

MicroRNAs (miRNAs) are a class of small noncoding RNAs that regulate gene expression at the posttranscriptional level. These noncoding RNAs have recently emerged as crucial regulators of aging process [15-17]. Emerging evidence has shown alteration in miRNA expression profile in the skeletal muscles of both human and mouse during aging [18-25]. For example, elevated let-7 family members in skeletal muscle contribute to reduced cellular proliferation and regenerative capacity in aged human [22]. Aging alters the expression of 57 miRNAs in mouse quadriceps muscles and few of them are associated with reduced cell proliferation and favored the terminal differentiation of myogenic precursor [20]. Further-more, resistance exercise, caloric restriction, or nutrientrelated hormones such as the adipokine leptin reverse the expression of age-regulated miRNAs [18, 21, 26]. Interestingly, it has been shown that miR-210 can mediate hypoxia-induced apoptosis of neuroblastoma cells by targeting expression of the anti-apoptotic protein Bcl-2 gene [27]. The above studies suggest the possibility that dysregulation of miRNAs in skeletal muscle during aging may induce sarcopenia by activating catabolic pathways, including apoptosis. However, no study to date has demonstrated a role for miRNA in skeletal muscle apoptosis, especially during aging. In the current study, we show that aging dysregulates many miRNAs in skeletal muscle, including the highly downregulated miRNA miR-434-3p that inhibits apoptosis by targeting eIF5A1 that promote apoptosis by the intrinsic mitochondrial pathway.

## RESULTS

### Aging dysregulates microRNA expression profile in skeletal muscle

**Figure 1:**
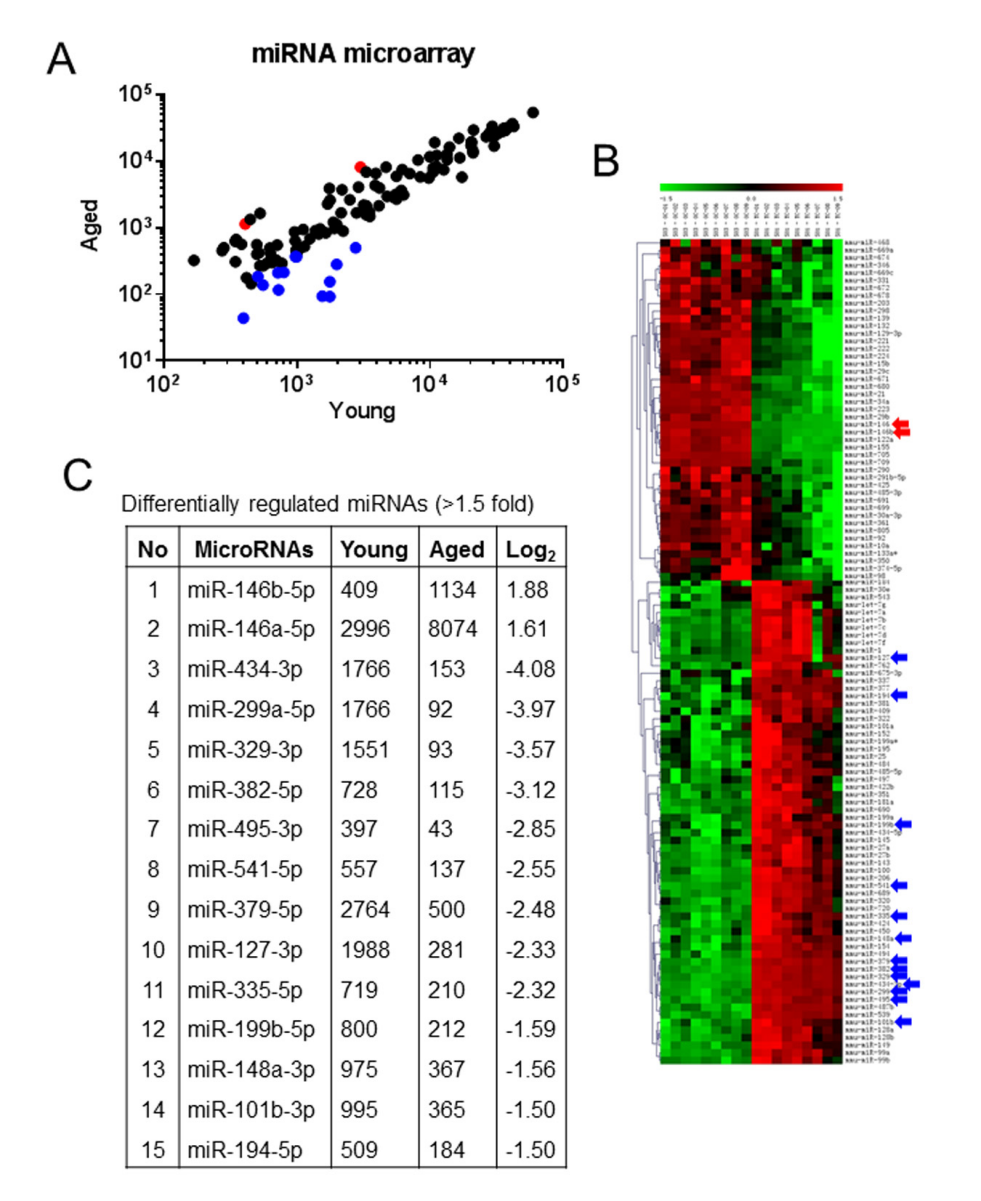
Aging alters miRNA expression profile in skeletal muscle (**A** and **B**) Total RNA was isolated from the skeletal muscles from three-month-old young control and 26-month-old aging mice and used in miRNA microarray analyses to determine the expression levels of mouse miRNAs. Data on the scatter plot shows log10-transformed signal intensities for each probe labeled with Cy3 (young) and Cy5 (aging) mice (**A**). The heat map shows miRNAs significantly differentially expressed in skeletal muscle from aging mice (**B**). Each dot represents one miRNA probe. (**C**) Differentially-regulated (≥ 1.5 fold) age-related miRNAs.

**Figure 2:**
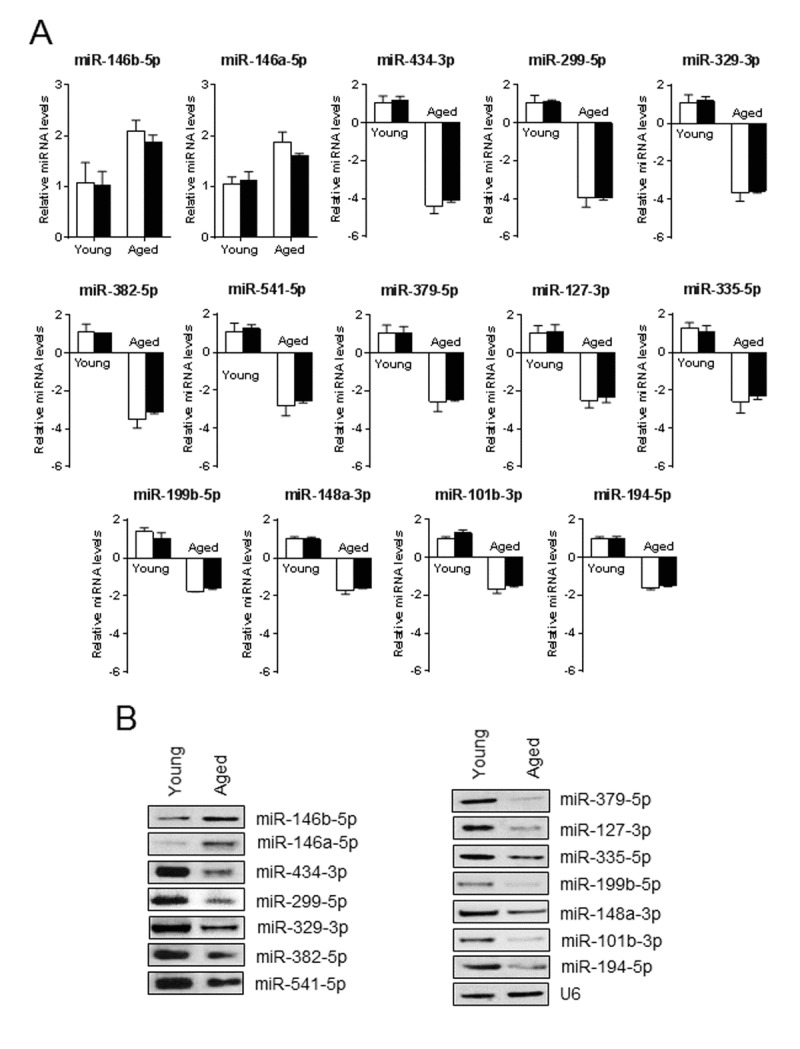
Validation of aging associated miRNA expression profile (**A** and **B**) A portion of RNA used in the microarray was used in qPCR (**A**) and in a separate experiment by solution hybridization technique with 5' biotin-labeled miRNAs (**B**) to confirm the expression level of miRNAs that were differentially regulated ≥1.5-fold in the microarray. U6 served as both loading control and normalizer. Gel pictures are representative of three independent experiments. Each bar indicates mean ± SEM (n = 3). White and black bars indicate the levels of miRNA measured by microarray and qPCR, respectively.

To explore the effect of aging in the regulation of miRNA expression profile in skeletal muscle, we performed a miRNA microarray screening using total RNA isolated from the gastrocnemius (GA) muscles of young and old mice. The array uncovered the induction of 117 miRNAs with the signal intensity ≥500 (the fluorescence amount of each miRNA probe is measured by a photo multiplier tube or charge-coupled device and signal scaled across the range of detection for the platform) in GA muscle (Table 1, Fig. 1A and 1B), including the highly downregulated miRNAs (≥1.5-fold) miR-194-5p, miR-101b-3p, miR-148a-3p, miR-199b-5p, miR-335-5p, miR-127-3p, miR-379-5p, miR-541-5p, miR-382-5p, miR-329-3p, miR-299-5p and miR-434-3p, and the highly up-regulated miRNAs (≥1.5 fold), miR-146b-5p and miR-146a-5p (Fig. 1C). Solution hybridization and real-time PCR assays confirmed the microarray findings (Fig. 2A and 2B). The small nuclear RNA U6, control, and a normalizer for miRNAs, was relatively unchanged and that excluded the possibility of artifactual changes in miRNA recovery.

**Table 1:** Differentially regulated miRNAs in the GA muscle of aging mouse

No	miRNA	Young	Old	Log_2_	No	miRNAs	Young	Old	Log_2_
1	miR-146b	410	1134	1.88	35	let-7f	35689	30911	−0.2
2	miR-146	2997	8074	1.61	36	let-7e	20397	19144	−0.2
3	miR-155	448	1338	1.28	37	let-7d	31845	27066	−0.21
4	miR-29b	345	626	1.16	38	let-7a	38019	31797	−0.23
5	miR-223	531	1646	0.99	39	miR-30d	8127	10391	−0.23
6	miR-671	384	565	0.74	40	miR-30b	29922	22576	−0.23
7	miR-705	4676	8084	0.71	41	let-7c	32084	25550	−0.25
8	miR-21	10838	19006	0.49	42	let-7b	29276	24279	−0.25
9	miR-203	350	661	0.48	43	miR-486	10521	7839	−0.27
10	miR-221	962	862	0.35	44	let-7g	26308	23201	−0.28
11	miR-709	29237	33168	0.29	45	miR-16	13343	12570	−0.28
12	miR-92	1753	3888	0.28	46	let-7i	20275	17125	−0.31
13	miR-29c	2183	3688	0.26	47	miR-133a*	1493	956	−0.31
14	miR-98	3911	6513	0.19	48	miR-125b	20431	19224	−0.31
15	miR-224	284	500	0.11	49	miR-30a-5p	11201	12191	−0.32
16	miR-805	3315	6839	0.1	50	miR-451	6331	3093	−0.34
17	miR-222	711	546	0.08	51	miR-139	1117	934	−0.34
18	miR-15b	2903	4074	0.05	52	miR-185	3866	4299	−0.35
19	miR-29a	28014	26006	0.03	53	miR-10b	4182	3948	−0.35
20	miR-133b	40419	33589	0.02	54	miR-191	6182	7454	−0.36
21	miR-126-5p	2498	2630	0.01	55	miR-24	9958	11637	−0.4
22	miR-23b	21187	29118	−0.06	56	miR-150	10549	6830	−0.4
23	miR-23a	36640	28636	−0.07	57	miR-214	8430	5777	−0.41
24	miR-361	1776	2579	−0.08	58	miR-103	3167	2176	−0.49
25	miR-1	59283	53604	−0.08	59	miR-27b	20914	14373	−0.5
26	miR-26b	28063	26507	−0.09	60	miR-27a	20998	13352	−0.52
27	miR-133a	34591	27564	−0.1	61	miR-195	16713	11172	−0.53
28	miR-125a	13997	16139	−0.1	62	miR-199a*	13393	10549	−0.53
29	miR-10a	5599	5923	−0.11	63	miR-22	7043	6474	−0.56
30	miR-26a	41407	36396	−0.11	64	miR-145	11134	8288	−0.62
31	miR-30c	16433	21901	−0.12	65	miR-107	3321	2128	−0.65
32	miR-132	500	554	−0.15	66	miR-422b	12684	7357	−0.66
33	miR-30a-3p	1703	2266	−0.17	67	miR-20a	1461	806	−0.67
34	miR-126-3p	42474	32921	−0.19	68	miR-15a	2152	1662	−0.69
34	miR-17-5p	520	420	−0.7	94	miR-181a	3521	1470	−1.14
36	miR-674	633	416	−0.7	95	miR-199a	994	405	−1.2
37	miR-320	5549	3168	−0.72	96	miR-62101a	1142	524	−1.22
38	miR-676	701	326	−0.73	97	miR-72630	1528	877	−1.25
39	miR-206	30292	16791	−0.76	98	miR-36564	500	400	−1.29
40	miR-151	973	634	−0.76	99	miR-30e65	1651	836	−1.29
41	miR-762	6082	3620	−0.78	100	miR-690	2803	1669	−1.3
42	miR-181b	1882	1210	−0.78	101	miR-322	773	291	−1.34
43	miR-143	9802	5554	−0.79	102	miR-424	3305	1568	−1.38
44	miR-25	4761	2932	−0.8	103	miR-128b	1989	1114	−1.44
45	miR-342	1915	970	−0.82	104	miR-128a	2230	885	−1.48
46	miR-378	988	459	−0.85	105	miR-689	17376	5739	−1.49
47	miR-152	4130	2123	−0.86	106	miR-194	510	184	−1.5
48	miR-130a	627	497	−0.87	107	miR-101b	995	366	−1.5
49	miR-140*	1250	683	−0.89	108	miR-148a	975	367	−1.56
50	miR-28	1060	493	−0.92	109	miR-199b	800	213	−1.59
51	miR-106b	598	293	−0.95	110	miR-335	720	210	−2.32
52	miR-99b	3513	1699	−0.98	111	miR-127	1989	282	−2.33
53	let-7d*	527	267	−1	112	miR-379	2765	500	−2.48
54	miR-328	652	300	−1	113	miR-541	557	137	−2.55
55	miR-99a	5591	2684	−1.01	114	miR-382	729	116	−3.12
56	miR-100	5263	2899	−1.03	115	miR-329	1551	93	−3.57
57	miR-499	1377	901	−1.04	116	miR-299	1767	92	−3.97
58	miR-181d	559	269	−1.05	117	miR-434-3p	1767	154	−4.08
59	miR-149	3221	1683	−1.06					

Underlines indicate differentially regulated miRNAs at signal intensities >10,000.

### Identification of predicted biological pathways of dysregulated-miRNAs in aging skeletal muscle

To determine predicted biological pathways of miRNAs differentially regulated (≥1.5 fold) in the aging skeletal muscle, we used DIANA miRPath v 3.0 software. The threshold p values for the pathway and MicroT were set to 0.05 and 0.8, respectively. The KEGG pathway in DIANA miRPath identified 87 (Table 2) biological pathways, including, 20 pathways for up-regulated miRNAs and 67 pathways for down-regulated miRNAs. Among those pathways, 7 (up-regulated miRNAs) and 25 (down-regulated miRNAs) pathways were associated with skeletal muscle functions (Table 3 and 4). According to gene ontology, there are 24 genes involved in the seven pathways, whereas 785 genes are involved in 25 pathways. The DIANA miRPath provided 8 predicted skeletal muscle pathways, including apoptosis for the highly downregulated miRNA-434-3p (Table 5).

**Table 2:** Predicted biological pathways linked to differentially regulated miRNAs (>1.5-fold) in the GA muscle of aging mouse

No	KEGG pathway	p-value	Genes	miRNAs
1	**PI3K-Akt signaling pathway**	**7.21E-12**	**90**	**13**
2	Pathways in cancer	1.79E-10	88	13
3	**MAPK signaling pathway**	**1.90E-20**	**82**	**14**
4	HTLV-I infection	6.23E-12	77	14
5	**Regulation of actin cytoskeleton**	**8.73E-13**	**65**	**14**
6	**Focal adhesion**	**8.92E-16**	**63**	**12**
7	**Wnt signaling pathway**	**3.09E-23**	**59**	**13**
8	Transcriptional misregulation in cancer	2.36E-11	58	14
9	Protein processing in endoplasmic reticulum	3.17E-12	53	12
10	Axon guidance	5.07E-23	52	13
11	Endocytosis	5.35E-05	51	13
12	Dopaminergic synapse	2.95E-15	48	10
13	**Ubiquitin mediated proteolysis**	**1.08E-10**	**45**	**12**
14	Chemokine signaling pathway	0.000375	45	14
15	**Insulin signaling pathway**	**3.53E-11**	**43**	**13**
16	Calcium signaling pathway	0.000783	43	12
17	Hepatitis B	1.72E-06	41	14
18	Neurotrophin signaling pathway	9.78E-12	40	13
19	Tuberculosis	0.003419	40	13
20	Glutamatergic synapse	3.53E-11	39	11
21	T cell receptor signaling pathway	1.38E-11	37	14
22	Cholinergic synapse	7.49E-07	36	11
23	RNA transport	0.021696	35	13
24	Retrograde endocannabinoid signaling	1.20E-07	34	9
25	ErbB signaling pathway	1.43E-14	33	12
26	Oocyte meiosis	9.73E-05	32	11
27	**Cell cycle**	**0.004448**	**32**	**13**
28	Prostate cancer	8.27E-12	31	13
29	Osteoclast differentiation	7.19E-05	31	11
30	Serotonergic synapse	0.005448	30	11
31	Chagas disease (American trypanosomiasis)	2.70E-05	29	13
32	Hepatitis C	0.027024	29	13
33	Spliceosome	0.027549	29	11
34	Chronic myeloid leukemia	4.29E-10	27	13
35	Hypertrophic cardiomyopathy (HCM)	1.32E-08	27	8
36	Renal cell carcinoma	8.82E-08	27	12
37	GnRH signaling pathway	5.84E-07	27	13
38	Melanogenesis	7.49E-05	27	11
39	GABAergic synapse	0.004621	27	9
40	Long-term potentiation	8.27E-12	26	11
41	Small cell lung cancer	6.84E-07	26	13
42	Dilated cardiomyopathy	3.62E-06	26	8
43	**Apoptosis**	**3.24E-05**	**26**	**12**
44	mRNA surveillance pathway	0.001512	26	12
45	Pancreatic cancer	2.37E-08	25	11
46	Melanoma	2.80E-08	25	11
47	B cell receptor signaling pathway	1.52E-07	25	13
48	**TGF-beta signaling pathway**	**4.55E-05**	**25**	**12**
49	**p53 signaling pathway**	**3.29E-07**	**23**	**10**
50	**Adherens junction**	**3.18E-05**	**23**	**11**
51	Arrhythmogenic right ventricular cardiomyopathy	0.000115	23	10
52	Progesterone-mediated oocyte maturation	0.000564	23	11
53	Amphetamine addiction	0.00381	23	12
54	**mTOR signaling pathway**	**1.20E-07**	**22**	**11**
55	**VEGF signaling pathway**	**1.20E-07**	**22**	**11**
56	Glioma	6.54E-05	22	12
57	Bacterial invasion of epithelial cells	7.19E-05	21	8
58	Salmonella infection	0.000308	21	9
59	**Phosphatidylinositol signaling system**	**0.005448**	**21**	**10**
60	**Adipocytokine signaling pathway**	**4.03E-05**	**20**	**11**
61	Gastric acid secretion	0.001232	20	9
62	Non-small cell lung cancer	2.32E-06	19	11
63	Colorectal cancer	0.000274	19	11
64	**Gap junction**	**0.00222**	**19**	**12**
65	Endometrial cancer	1.79E-06	18	10
66	**Lysine degradation**	**3.31E-07**	**17**	**9**
67	Acute myeloid leukemia	3.07E-05	17	13
68	Amyotrophic lateral sclerosis (ALS)	9.65E-05	17	11
69	**Inositol phosphate metabolism**	**0.000552**	**17**	**10**
70	RIG-I-like receptor signaling pathway	0.01547	17	13
71	Fc epsilon RI signaling pathway	0.022008	17	10
72	**Hedgehog signaling pathway**	**2.88E-05**	**16**	**8**
73	**Calcium reabsorption**	**0.005096**	**16**	**9**
74	**Type II diabetes mellitus**	**0.00052**	**15**	**8**
75	Circadian rhythm	5.32E-08	14	7
76	Bladder cancer	0.001543	13	9
77	Basal transcription factors	0.002387	13	11
78	**Sphingolipid metabolism**	**0.028141**	**13**	**7**
79	Aldosterone-regulated sodium reabsorption	0.001227	12	11
80	Vasopressin-regulated water reabsorption	0.045885	12	6
81	Dorso-ventral axis formation	1.52E-07	10	10
82	**Alanine, aspartate and glutamate metabolism**	**0.016589**	**10**	**7**
83	Prion diseases	3.96E-10	7	7
84	Proximal tubule bicarbonate reclamation	0.004531	7	5
85	Taurine and hypotaurine metabolism	0.004447	4	3
86	D-Glutamine and D-glutamate metabolism	0.000519	3	3
87	**Biotin metabolism**	**0.00381**	**1**	**1**

Bold indicates skeletal muscle specific pathways.

**Table 3:** Predicted biological pathways controlled by up-regulated miRNAs

No	KEGG pathway	p-value	Genes	miRNAs
1	MAPK signaling pathway	0.028946	6	2
2	NF-kappa B signaling pathway	3.96E-05	4	2
3	Apoptosis	0.010616	4	2
4	Toll-like receptor signaling pathway	0.001913	3	2
5	VEGF signaling pathway	0.01676	3	2
6	ECM-receptor interaction	0.004068	2	2
7	TGF-beta signaling pathway	0.029883	2	2

**Table 4:** Predicted biological pathways controlled by downregulated miRNAs

No	KEGG pathway	p-value	Genes	miRNAs
1	PI3K-Akt signaling pathway	7.21E-12	90	13
2	MAPK signaling pathway	1.90E-20	82	14
3	Regulation of actin cytoskeleton	8.73E-13	65	14
4	Focal adhesion	8.92E-16	63	12
5	Wnt signaling pathway	3.09E-23	59	13
6	Ubiquitin mediated proteolysis	1.08E-10	45	12
7	Insulin signaling pathway	3.53E-11	43	13
8	Cell cycle	0.004448	32	13
9	Apoptosis	3.24E-05	26	12
10	TGF-beta signaling pathway	4.55E-05	25	12
11	Adherens junction	3.18E-05	23	11
12	p53 signaling pathway	3.29E-07	23	10
13	mTOR signaling pathway	1.20E-07	22	11
14	VEGF signaling pathway	1.20E-07	22	11
15	Phosphatidylinositol signaling system	0.005448	21	10
16	Adipocytokine signaling pathway	4.03E-05	20	11
17	Gap junction	0.00222	19	12
18	Inositol phosphate metabolism	0.000552	17	10
19	Lysine degradation	3.31E-07	17	9
20	Calcium reabsorption	0.005096	16	9
21	Hedgehog signaling pathway	2.88E-05	16	8
22	Type II diabetes mellitus	0.00052	15	8
23	Sphingolipid metabolism	0.028141	13	7
24	Alanine, aspartate and glutamate metabolism	0.016589	10	7
25	Biotin metabolism	0.00381	1	1

**Table 5:** Predicted biological pathways controlled by miR-434-3p

No	KEGG pathway	p-value	Genes	miRNAs
1	Fatty acid degradation	2.65E-10	1	1
2	Fatty acid metabolism	2.58E-06	1	1
3	Sphingolipid metabolism	0.002379	3	1
4	MAPK signaling pathway	0.002885	10	1
5	N-Glycan biosynthesis	0.003339	3	1
6	Valine, leucine and isoleucine degradation	0.005375	1	1
7	Apoptosis	0.010371	3	1
8	PI3K-Akt signaling pathway	0.021814	7	1

### eIF5A1 is a target mRNA of miR-434-3p

**Figure 3:**
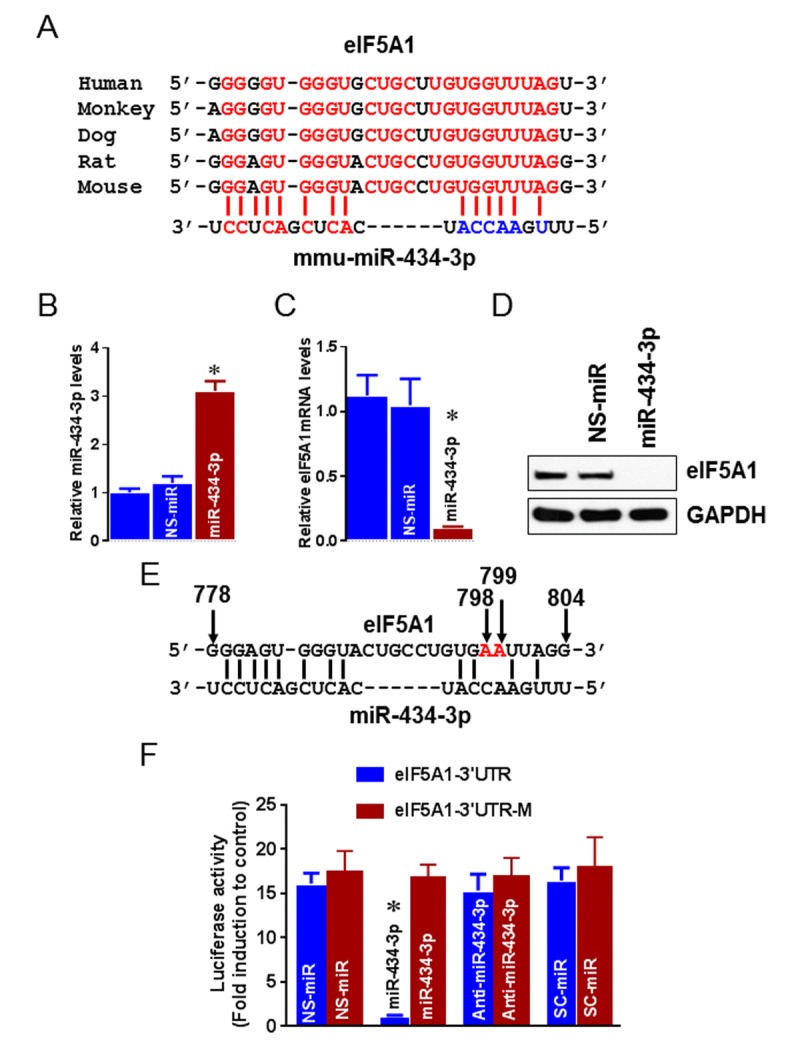
eIF5A1 is a target mRNA of miR-434-3p (**A**) Sequence alignment of putative miR-434-3p targeting site in the 3′-UTR of eIF5A1 shows high levels of complementarity. (**B**-**D**) Myotubes were transfected with miR-434-3p mimetic or NS-mimetic. miR-434-3p overexpression was determined by qPCR assay (B). U6 served as a normalizer. eIF5A1 mRNA levels were analyzed 24 h after transfection by qPCR (**C**) and eIF5A1 protein levels were analyzed 36 h after transfection by Western blot (**D**). (**E**) Sequence information represents the site directed mutagenesis on the 3′UTR of eIF5A1. Red letters indicate mutated nucleotide and arrows indicate nucleotide position. (**F**) The eIF5A1 3′-UTR–luciferase construct or eIF5A1 3′-UTR mutated–luciferase construct were co-transfected with NS-mimetic, miR-434-3p mimetic, miR-434-3p antagomir or SC-miR. Forty-eight hours after transfection, cells were collected, and then firefly luciferase activities were estimated and normalized to Renilla luciferase activities. Each bar indicates mean ± SEM (n = 3). *P < 0.05 vs. NS-miR. Gel pictures are representative of three independent experiments.

Because miR-434-3p is the highly downregulated miRNA in the skeletal muscle of aging mice, we searched for predicted miR-434-3p target genes using the public database of RNA22 [28]. Interestingly, the database listed eIF5A1 as one of the potential targets of miR-434-3p. Moreover, eIF5A1 has a conservative miR-434-3p seed sequence in its 3′-UTR (Fig. 3A). These data provided a strong rationale to test the possibility that eIF5A1 may be a downstream target of miR-434-3p. First, we tested whether miR-434-3p transcriptionally or post-transcriptionally suppresses endogenous eIF5A1 expression. To test this possibility, we transfected myotubes with NS-miR (non-specific miRNA) or miR-434-3p mimic. Myotubes carrying miR-434-3p showed overexpression of miR-434-3p by approximately three-fold (Fig. 3B). Enforced expression of miR-434-3p significantly decreased eIF5A1 mRNA levels (Fig. 3C). The reduction in eIF5A1 mRNA levels was concomitant with a decrease in eIF5A1 protein levels (Fig. 3D), suggesting that miR-149 predominantly suppresses eIF5A1 mRNA levels. Subsequently, we tested that whether eIF5A1 3′UTR is directly targeted by miR-434-3p. A reporter construct containing the luciferase gene fused to the eIF5A1 3′-UTR (luc-eIF5A1-3′-UTR) or luciferase gene fused to the mutated eIF5A1 3′-UTR (luc-eIF5A1-3′-UTR-M) (Fig. 3E) was co-transfected with NS-miR or miR-434-3p mimics with or without miR-434-3p antagomir into myotubes. As shown in Fig. 3F, while cells transfected with luc-eIF5A1-3′-UTR alone or co-transfected with NS-miR had luciferase activity, cells co-transfected with miR-434-3p mimics displayed a reduction in luciferase activity significantly. The transfection of miR-434-3p antagomir along with luc-eIF5A1-3′-UTR and miR-434-3p mimics restored the luciferase activity. Surprisingly, miR-434-3p and eIF5a1 3′UTR binding is only by six base pair matching that may cause a weaker interaction. Therefore, we mutated the miR-434-3p binding site on eIF5A1-3′-UTR to determine whether eIF5A1 is a direct target of miR-434-3p. Our luciferase assay experiments with mutations in the binding sites clearly confirmed eIF5a1 is a direct target mRNA of miR-434-3p (Fig. 3F). Overall, these data provide experimental evidence that eIF5A1 is a direct target gene of miR-434-3p.

### miR-434-3p protects myocytes from apoptosis through eIF5A1

**Figure 4:**
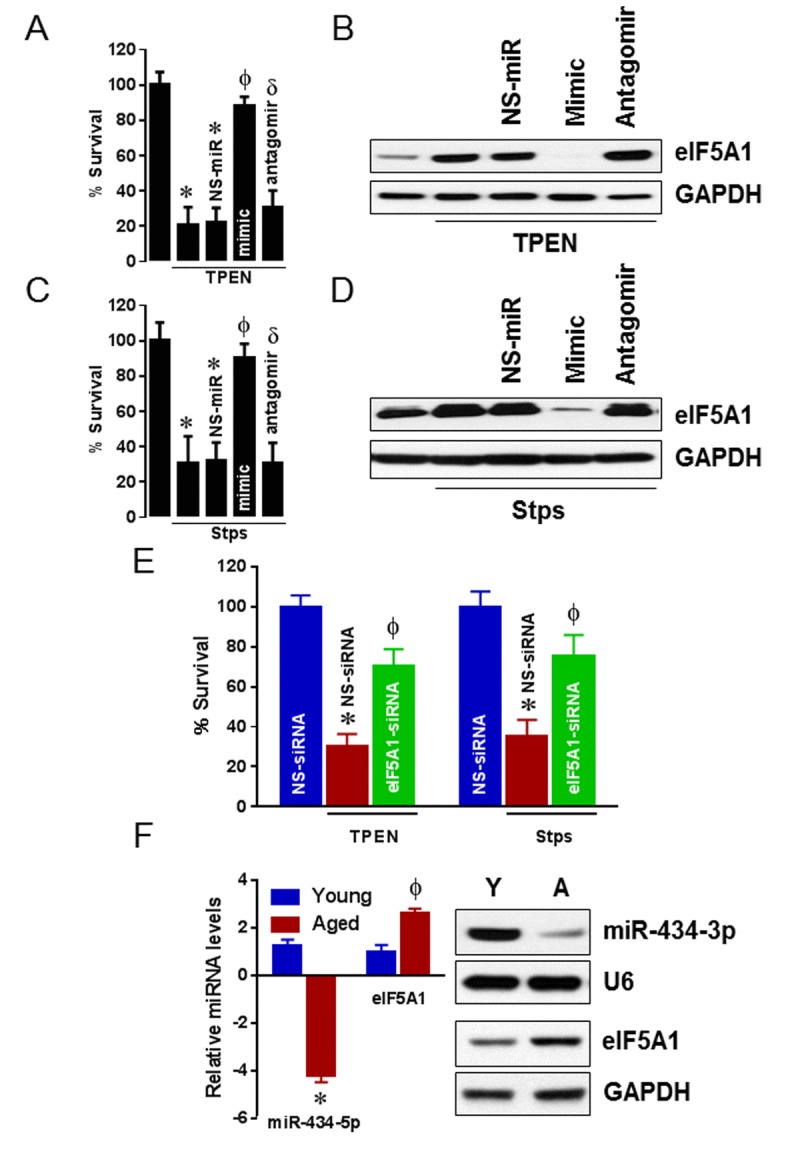
miR-434-3p protects apoptosis through eIF5A1 Myotubes were transfected with NS-mimetic, miR-434-3p mimetic with or without miR-434-3p antagomir or no transfection for 24 h followed by eight hours TPEN (100 μM) or Stps (1 μM) incubation. (**A** and **B**) Apoptosis (%) was measured by MTT assay in TPEN treated and non-treated myotubes (**A**) and the expression of eIF5A1 protein levels were determined by western blot (**B**). (**C** and **D**) Apoptosis (%) was measured by MTT assay in Stps treated and non-treated myotubes (**C**) and the expression of eIF5A1 protein levels were determined by western blot (**D**). (**E**) Myotubes were transfected with non-specific siRNA (NS-siRNA) or eIF5A1 siRNA for 24 h followed by eight hours TPEN (100 μM) or Stps (1 μM) incubation. Apoptosis (%) was measured by MTT assay. (**F**) miR-434-3p and eIF5A1 levels in young and aging GA muscles were determined by solution hybridization and western blot methods, respectively. U6 and GAPDH were served as loading controls for miR-434-3p and eIF5A1, respectively. Gel pictures are representative of three independent experiments. Each bar indicates mean ± SEM (n = 3). *, p< 0.05 vs. control; ϕ, p <0.05 vs. NS-miR or NS-siRNA; δ, p < 0.05 vs. mimic.

Previous studies have shown that eIF5A1 promotes apoptosis in a variety of cells [29-32]. Our prediction pathway analysis for miR-434-3p identified apoptosis as one of the skeletal muscle-specific pathways. Because eIF5A1 is a direct downstream target of miR-434-3p, we sought to determine if modulation of miR-434-3p would control apoptosis through eIF5A1 in myotubes. TPEN (a potent inducer of apoptosis) has been shown to induce apoptosis in myocytes [33]. Myotubes were transfected with miR-434-3p mimics or NS-miR for 36 h before treatment with TPEN for 24 h. Our data show that myotubes transfected with NS-miRNA before TPEN exposure had no protection against TPEN-induced apoptosis (Fig. 4A). In contrast, myotubes transfected with miR-434-3p mimics before TPEN treatment had an about 70% reduction in the percentage of apoptotic cells compared with myotubes transfected with NS-miRNA mimics. The transfection of miR-434-3p antagomir along with miR-434-3p mimics restored the TPEN-induced myotubes death (Fig. 4A). Western blot analysis indicated that TPEN-treated myotubes displayed an increased levels of eIF5A1 protein whereas myotubes transfected with NS-miR or myotubes that were not transfected had no effect on myotubes survival. On the other hand, transfection of myotubes with miR-434-3p mimics strongly reduced eIF5A1 protein levels and the cotransfection of myotubes with miR-434-3p antagomir reinstated eIF5A1 protein levels (Fig. 4B). To further confirm the role of miR-434-3p in apoptosis, we used staurosporine (Stsp), a well-known transcription inhibitors, has been shown to induce apoptosis in several cell types including myocytes [33, 34]. While myotubes treated with Stsp had a higher percentage of death, transfection of those myotubes with miR-434-3p mimic increased the proportion of myotubes survival and co-transfection of myotubes with miR-434-3p antagomir reversed the effect of miR-434-mimic (Fig. 4C). Furthermore, loss of myotubes survival due to Stsp treatment correlated with a significant increase in the levels of eIF5A1 protein. Such altered expression of elF5A1 was reduced when those myotubes were transfected with miR-434-3p antagomir (Fig. 4D). In addition, knockdown of eIF5A1 by siRNA in myotubes significantly reduced the TPEN or Stsp -induced cell death (Fig. 4E), suggesting that eIF5A1 is directly responsible for the TPEN or Stsp-induced apoptotic effect. Furthermore, we found that GA muscle from aging mice displayed lower expression levels of miR-434-3p when compared to that in the GA muscle of young mice (Fig. 4F). In contrast, the levels of eIF5A1 mRNA and protein were higher in the GA muscle of aging mice when compared to that in the GA muscle of young mice (Fig. 4F). These data confirm that miR-434-3p protects myocyte from apoptosis induced by different apoptotic stimuli *via* eIF5A1 and a negative correlation between the expression of miR-434-3p and eIF5A1 in aging muscle.

### Overexpression of miR-434-3p reduces activation of caspases 3, 8, and 9 in myotubes

**Figure 5:**
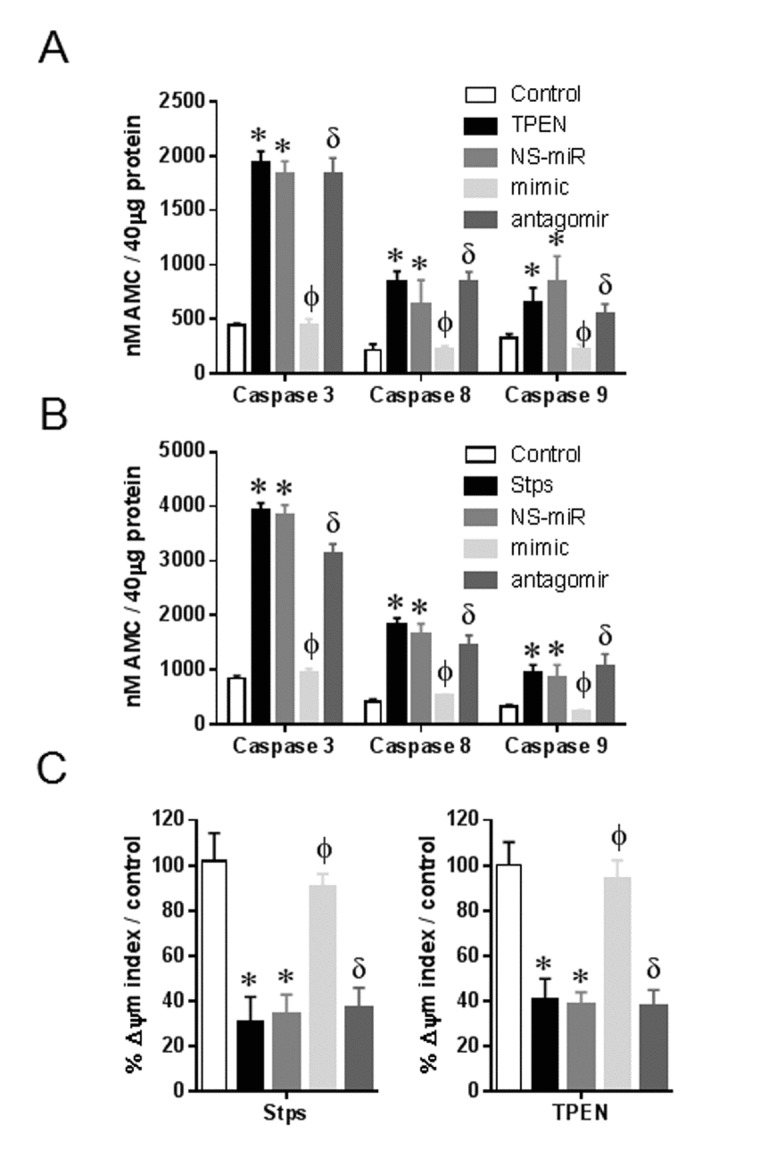
miR-434-3p protects myotubes from caspase activation and loss of mitochondrial membrane potential Myotubes were transfected with NS-mimetic, miR-434-3p mimetic, miR-434-3p antagomir or no transfection for 24 h followed by eight hours TPEN (100 μM) or Stps (1 μM) incubation. (**A** and **B**) Enzymatic activities of caspase-3, −8 and −9 in myotubes treated with TPEN (A) and Stsp (B) were determined by caspase specific fluorogenic substrates. (**C**) Untreated (control) or treated myotubes were stained with JC-1 and observed under TRITC (590 nm) and GFP (530 nm) filters. %Δψm index represents the ratio of red to green fluorescence. Each bar indicates mean ± SEM (n = 3). *, p < 0.05 vs. control; ϕ, p < 0.05 vs. NS-miR; δ, p < 0.05 vs. mimic.

To determine whether miR-434-3p-regulated apoptosis involves inhibition of activation of caspases, the effects of their over-expression on the activation of executioner caspase-3 as well as initiator caspases −8 and −9 were examined in myotubes over a period of 24 h. All three caspases were activated in response to TPEN or Stps treatment; however, caspase 3 activity was dramatically higher when compared to caspases 8 and 9, especially in Stps treated myotubes (Fig. 5A and B). Myotubes transfected with miR-434-3p mimic significantly suppressed all three caspases activities, and the cotransfection of miR-434-antagomir restored the TPEN or Stps-induced caspases activities whereas myotubes transfected with NS-miR had no effect on all three caspases activities in response to TPEN or Stps treatment (Fig. 6A and B). One of the earliest events in the progression of apoptosis is the dissipation of the mito-chondrial membrane potential (∆ψm). Our data in Fig 5C and D showed suppression of caspases 3, 8 and 9 activities by miR-434-3p, suggesting the possibility that miR-434-3p may maintain Δψm. JC-1 is a different cationic dye has been used to measure the collapse of the electrochemical gradient across the mitochondrial membrane [34, 35]. In normal untreated myotubes, JC-1 is present as a red fluorescent aggregate in mito-chondria, and in the green fluorescent monomeric form in the cytosol in myotubes. Upon the treatment with TPEN or Stsp, there is a dissipation of the ∆ψm indicated by the increase in staining in the green filter (530 nm) and a decrease in staining in the red filter (590 nm) in myotubes. Quantification of the fluorescent intensities indicated that myotubes treated with TPEN or Stsp significantly decreased the ∆ψm index while myotubes transfected with miR-434-3p mimic reversed the scenario; in contrast, the cotransfection of antagomir restored the effect of TPEN and Stsp in ∆ψm (Fig. 5C). These data suggest that the diminished depolarization of mitochondrial membrane potential of myotubes in response to TPEN and Stsp may be due to suppression of apoptosis by miR-434-3p through eIF5A1.

## DISCUSSION

Sarcopenia is a progressive decline in skeletal muscle mass, strength, and quality during aging. Although the molecular mechanisms involved in sarcopenia development are not entirely understood, it is welldocumented that transcriptomic, proteomic and epigenetic changes during the progression of aging are the main causative factors for sarcopenia. These epigenetic changes could increase oxidative stress and *vice versa* and lead to muscle loss and function due to activation of multiple catabolic pathways, including apoptosis. The main objective of this study was to determine the effect of aging on miRNA expression profile and identify how dysregulation of a specific miRNA in aging muscle could cause the activation of the catabolic pathway(s). Using microarray analysis, we found that aging dysregulated miRNA expression profile in skeletal muscle and most of the dysregulated miRNAs are suppressed in aging muscle. Using extensive bioinformatics analysis, we identified the eukaryotic translation initiation factor 5A1 (eIF5A1) as one of the potential target genes of the most highly downregulated miRNA miR-434-3p. Overexpression or knockdown of miR-434-3p in myotubes validated eIF5A1 as a target mRNA of miR-434-3p that negatively regulated apoptosis through eIFA1. Further-more, dual-luciferase assay validation method further confirmed miR-434-3p binding site within the 3′UTR mRNA encoding eIF5A1. Interestingly, we found that skeletal muscle from aging mice downregulated of miR-434-3p expression that was negatively correlated with the levels of eIF5A1, suggesting that dysregulation of miR-434-3p in aging muscle may be responsible, in part, for the pathogenicity of sarcopenia.

In the present study, we found that aging dysregulated many miRNAs, including two upregulated and 13 downregulated (≥1.5 fold) miRNAs in skeletal muscle, suggesting diminished miRNA expressions profile in aging muscle, and this is in agreement with previous findings [19, 23, 25, 36]. The differentially expressed miRNAs in earlier studies appeared consistently in mouse skeletal muscle from our study; for example, the expression pattern of miR-146a-5p, miR-146b-5p, miR-434-3p, miR-127-3p, and miR-148a-3p are similar to previous studies. In our study, we found that miR-434-3p was the highly downregulated mRNA (4.08 fold) in the aging muscle; this is also in agreement with the previous study in which aging mice downregulates this miRNA at 5.0 fold in skeletal muscle [20]. This suggesting that miR-434-3p may have a crucial role in the etiology of aging. Using extensive bioinformatics analysis, we identified eIF5A1 as one of the potential target genes of miR-434-3p. We confirmed that miR-434-3p indeed suppressed eIF5A1 mRNA expression by binding on the 3′UTR of eIF5A1, as evidenced by western blot analysis and luciferase assay. Moreover, we for the first time demonstrate that aging muscle up-regulated eIF5A1 protein expression, suggesting a negative correlation in the expression levels between miR-434-3p and eIF5A1 in the aging muscle. In agreement with our findings, a previous study demonstrates that overexpression of miR-434-3p *in vivo* rat skeletal muscle post-transcriptionally suppresses eIF5A1 expression and both miR-434-3p and eIR5A1 are regulated in muscle in an opposite manner after spinal cord injury [37]. Although miR-434-3p was the most highly downregulated miRNA in the skeletal muscle of aged mice, the signal intensity was relatively small (1767) when compare to other miRNAs such as miR-1 (59283) and miR-126-3p (42474) as evidenced by microarray that is not a quantitative assay and signal intensity more than 500 is biologically relevant. Interestingly, our quantitative PCR assay in Figures 2A and 4F show that miR-434-3p was nearly 4-fold significantly downregulated in aging muscle when compared to that in the muscle of young mice, suggesting that aging significantly affected the expression of miR-434-3p. This significance was confirmed by solution hybridization detection (miR-434-3p) and western blot (eIF5A1). In addition, the miR-434-3p is absence in human according to miRBase 21 that limits the translational significance. Because many miRNAs can target a single mRNA, other human miRNA(s) that target eIF5A1 may have a similar role of miR-434-3p and that specific miRNA(s) may reinstate the translational significance of miR-434-3p. Overall our findings suggest that the dysregulation of the miR-434-3p/eIF5A1 pathway in aging muscle may play a role in the pathogenicity of sarcopenia.

The small protein eIF5A1 promotes the initiation and translation elongation phases of protein synthesis by transient association with 80S ribosome complex [38-41]. However, numerous other functions have also been identified for eIF5A, including apoptosis in a variety of cells [31, 32, 42]. In this study, up-regulation of eIF5A1 protein and downregulation of miR-434-3p in aging skeletal muscle suggest that miR-434-3p/eIF5A1 pathway may induce apoptosis in skeletal muscle during aging and that may be one of the epigenetic causative mechanisms for the induction of sarcopenia. Although the etiology of sarcopenia is complex and characterized by the contribution of multiple factors [43], there is growing evidence for a prominent role of accelerated apoptosis in sarcopenia [12, 43-47].

Previous studies identified a role for eIF5A1 in apoptosis; these studies demonstrate that siRNA-mediated suppression of eIF5A1 expression protects cells against apoptosis induced by TNF-α [30], Actinomycin D [31, 32], sodium nitroprusside as well as the proteasome inhibitor MG-132 [32]. Conversely, over-expression of eIF5A1 has been shown to induce apoptosis [31, 32, 42], further supporting the apoptogenic nature of eIF5A1. This study sheds some light to determine how eIF5A1 engages in apoptosis in skeletal muscle during aging. We found that eIF5A1 facilitates activation of the intrinsic mitochondrial apoptotic pathway. We demonstrate that knockdown of miR-434-3p in myotubes induced activation of caspase-3, −8 and −9, and permeabilization of the outer mitochondrial membrane along with up-regulation of eIF5A1, suggesting that loss of miR-434-3p promotes apoptosis by facilitating activation of the intrinsic mitochondrial pathway. We confirmed the above data by demonstrating that overexpression of miR-434-3p resulted in inhibition of caspase-3, −8 and −9 activations, and permeabilization of the outer mitochondrial membrane along with downregulation of eIF5A1. Oxidative damage to lipids, proteins, and DNA, especially in post-mitotic tissue like the skeletal muscle of an aged organism, may be severe and ultimately lead to apoptotic or necrotic cell death. We and others have previously shown an elevation of oxidative and apoptosis markers in skeletal muscle from aging mice [48-52]. Future studies should focus on determining the causal mechanism through which aging of skeletal muscles suppresses miR-434p.

## METHODS

### Animals

The Institutional Animal Care and Use Committee from the Baylor College of Medicine approved all experimental procedures. We used a total of 44 mice to complete the experiments mentioned in this study. These include three-month-old young (n=20), and 26 months old aged (n=24) male C57BL/6J mice (The Jackson Laboratory, Bar Harbor, ME). All mice were kept in a temperature-controlled room on a 12-h light/dark cycle, with a temperature of 23°C, humidity of 40–60%, and food and water ad libitum.

### Cell culture

For in vitro studies, we used primary myoblasts isolated from young mouse hind limb muscles and cultured them to induce myotubes as described previously [53].

### miRNA microarray analysis

We isolated total RNA from the skeletal muscles of young and aged mice and used for microarray analysis as mentioned earlier [54, 55]. The fold difference values of altered miRNAs were converted into log_2_ scale.

### Gene functional analysis

To identify the predicted biological pathways regulated by miRNAs (≥1.5-fold), we used the DIANA miRPath v2.0 Web-based computational tool with a threshold p-value of 0.05 and a MicroT threshold value of 0.8 [56]. miRNAs and their predicted targets were identified using the miRWalk Web-based computational tool, which provides miRNA targets from at least eight established miRNA prediction programs [57].

### Reverse transcription and quantitative PCR (RT-qPCR)

To validate differentially regulated miRNA data from microarray analysis, we performed miRNA RT-qPCR array as described earlier [54, 55]. To normalize mRNA and miRNA levels in qPCR, we used glyceraldehyde-3-phosphate dehydrogenase (GAPDH) and U6, respectively.

### Solution hybridization detection analysis

To confirm microarray data, we measured the expression levels of mature miRNAs by a solution hybridization detection method with mirVana miRNA and Bright-Star BioDetect kits as described previously [54].

### Western blot

Western blots were performed as documented earlier [53]. eIF5A1 and GAPDH antibodies were purchased from Cell Signaling, Danvers, MA.

### Transfection

Primary myoblasts were plated in 6-well plates (for immunoblot analyses) or 24-well plates (for 3-(4,5-dimethylthiazol-2-yl)-2,5-diphenyltetrazolium bromide [MTT] assay) in medium containing 10% FBS and allowed to adhere for 24 hours. The next day, myoblast differentiation was induced for three days, and the myotubes were transfected with 30nM of miR-434-3p mimetic or the NS microRNA (miR-NS) control (miRvana; Life Technologies). For RNAi study, the myotubes were transfected with 50 pmols NS-siRNA or eIF5A1-siRNA acoording to the manufacturer protocol (SantaCruz Biotechnology, Inc). All transfections were performed with Lipofectamine RNAi MAX (Life Technologies) according to the instructions provided by the manufacturer. The microRNA mimetic or siRNA and Lipofectamine solutions were prepared in Opti-MEM I Reduced Serum Medium (Life Technologies), mixed gently, and incubated 5–10 minutes at room temperature. The lipid-complexed microRNA mimetic was added gently to the 6- or 24-well plates and incubated as indicated in each experiment. Luciferase assays were performed as described previously [55]. Briefly, 2.5 mg of expression vector bearing mmu–miR-149 precursor, mouse pcDNA–PARP-2, mouse pcDNA–PARP-2 without 39-UTR, 2.3 mg of pmirGLO-PARP-2–39-UTR, or 400 ng of mmu–miR-149 miRCURY LNA knockdown probe (antagomir) or scrambled probe (Exiqon, Woburn, MA) was added.

### eIF5A1 3′-UTR mutagenesis

The miR-434-3p binding sites on eIF5A1 3′UTR were mutated as we described previously [58] using QuikChange II site-directed mutagenesis kit (Stratagene). Briefly, the eIF5A1 3′UTR at the miR-434-3p canonical binding sites, were obtained by replacing the 5′-GCCTGUGGTTTAGG-3 consensus sequence by 5′-GCCTGTGAATTAGG-3. The eIF5A1-3′ UTR was mutagenized by site-directed mutagenesis by using the following primers: −5′-TGCTTGTGGTTTAGGTTCCC-3′ and 5′-ATCGGGGATGAGTAGGATAA −3′ for eIF5A1-3′UTR-M.

### Induction of apoptosis

Apoptosis was induced in myotubes by treated with TPEN or staurosporine (Stsp) (Santa Cruz Biotechnology, Inc. Dallas, TX). Briefly, cells were treated for seven hours with TPEN (10 μM – 100 μM) or Stsp (0.5–2 μM) to induce apoptosis. We observed that about a 50–60% cell death occurred at 100 μM TPEN or 1 μM Stsp at eight hours (data not shown) and therefore we used this concentration for further experiments.

### Cell viability assay

Cell survival was measured using the standard MTT cell viability assay protocol. Briefly, primary myoblasts were plated in 24-well plates in medium containing 10% FBS and allowed to adhere for 24 hours. Then, the differentiation of primary myoblasts was induced as described previously [53]. After treatment of myotubes, cell viability was quantified by MTT (obtained from Sigma-Aldrich) and expressed as a percentage as mentioned earlier [59]. All experiments were repeated at least three times, with each experimental condition repeated at least in triplicate per experiment.

### Assessment of mitochondrial membrane potential (Δψm)

Because a decline in Δψm causes the escape of pro-apoptotic proteins that regulate both caspase-dependent and independent apoptosis from mitochondria into the cytosol, we measured Δψm as an early event in the initiation of apoptosis. After appropriate treatments of myotubes, Δψm was assessed using the cationic dye, JC-1 (5,5′, 6,6′-tetrachloro-1,1′,3,3′-tetraethyl-benzimidazolylcarbocyanine iodide). JC1 stains the healthy myotubes with bright red due to accumulation of the dye within the mitochondria whereas the dye stains the apoptotic myotubes with green because of the collapse of the mitochondrial membrane potential resulting in the dye unable to accumulate within the mitochondria. After treatment, myotubes were rinsed with PBS, followed by incubation with JC-1 reagent 1:100 at 37°C for 30 min. After rinsed myotubes twice with PBS, images were obtained using TRITC (red, 590 nm) and GFP (green, 530 nm) filters on a fluorescent microscope (Carl Zeiss). Care was taken to obtain pictures with identical exposure times, and pictures were analyzed using Carl Zeiss software. The automatic measurement program with same user defined parameters for densitometric and geometric variables was used to determine fluorescent intensity for both filters. The ratio of the sum of intensities of red over green fluorescence was identified and expressed as the Δψm index.

## References

[1] Rosenberg IH (1997). Sarcopenia: origins and clinical relevance. J Nutr.

[2] Fielding RA, Vellas B, Evans WJ, Bhasin S, Morley JE, Newman AB, Abellan van Kan G, Andrieu S, Bauer J, Breuille D, Cederholm T, Chandler J, De Meynard C, International Working Group on Sarcopenia (2011). Sarcopenia: an undiagnosed condition in older adults. Current consensus definition: prevalence, etiology, and consequences. International working group on sarcopenia. J Am Med Dir Assoc.

[3] Welch AA, MacGregor AJ, Minihane AM, Skinner J, Valdes AA, Spector TD, Cassidy A (2014). Dietary fat and fatty acid profile are associated with indices of skeletal muscle mass in women aged 18-79 years. J Nutr.

[4] Ghosh S, Lertwattanarak R, Garduno JD, Galeana JJ, Li J, Zamarripa F, Lancaster JL, Mohan S, Hussey S, Musi N (2015). Elevated Muscle TLR4 Expression and Metabolic Endotoxemia in Human Aging. J Gerontol A Biol Sci Med Sci.

[5] Jang YC, Sinha M, Cerletti M, Dall'Osso C, Wagers AJ (2011). Skeletal muscle stem cells: effects of aging and metabolism on muscle regenerative function. Cold Spring Harb Symp Quant Biol.

[6] Sousa-Victor P, Gutarra S, García-Prat L, Rodriguez-Ubreva J, Ortet L, Ruiz-Bonilla V, Jardí M, Ballestar E, González S, Serrano AL, Perdiguero E, Muñoz-Cánoves P (2014). Geriatric muscle stem cells switch reversible quiescence into senescence. Nature.

[7] García-Prat L, Sousa-Victor P, Muñoz-Cánoves P (2013). Functional dysregulation of stem cells during aging: a focus on skeletal muscle stem cells. FEBS J.

[8] Leeuwenburgh C (2003). Role of apoptosis in sarcopenia. J Gerontol A Biol Sci Med Sci.

[9] Meng SJ, Yu LJ (2010). Oxidative stress, molecular inflammation and sarcopenia. Int J Mol Sci.

[10] Alway SE, Degens H, Krishnamurthy G, Smith CA (2002). Potential role for Id myogenic repressors in apoptosis and attenuation of hypertrophy in muscles of aged rats. Am J Physiol Cell Physiol.

[11] Siu PM, Pistilli EE, Butler DC, Alway SE (2005). Aging influences cellular and molecular responses of apoptosis to skeletal muscle unloading. Am J Physiol Cell Physiol.

[12] Pistilli EE, Siu PM, Alway SE (2006). Molecular regulation of apoptosis in fast plantaris muscles of aged rats. J Gerontol A Biol Sci Med Sci.

[13] Alway SE, Siu PM (2008). Nuclear apoptosis contributes to sarcopenia. Exerc Sport Sci Rev.

[14] Phillips T, Leeuwenburgh C (2005). Muscle fiber specific apoptosis and TNF-alpha signaling in sarcopenia are attenuated by life-long calorie restriction. FASEB J.

[15] Lanceta J, Prough RA, Liang R, Wang E (2010). MicroRNA group disorganization in aging. Exp Gerontol.

[16] Jung HJ, Suh Y (2012). MicroRNA in Aging: From Discovery to Biology. Curr Genomics.

[17] Hodzic M, Naaldijk Y, Stolzing A (2013). Regulating aging in adult stem cells with microRNA. Z Gerontol Geriatr.

[18] Drummond MJ, McCarthy JJ, Fry CS, Esser KA, Rasmussen BB (2008). Aging differentially affects human skeletal muscle microRNA expression at rest and after an anabolic stimulus of resistance exercise and essential amino acids. Am J Physiol Endocrinol Metab.

[19] Kim JY, Park YK, Lee KP, Lee SM, Kang TW, Kim HJ, Dho SH, Kim SY, Kwon KS (2014). Genome-wide profiling of the microRNA-mRNA regulatory network in skeletal muscle with aging. Aging (Albany NY).

[20] Hamrick MW, Herberg S, Arounleut P, He HZ, Shiver A, Qi RQ, Zhou L, Isales CM, Mi QS (2010). The adipokine leptin increases skeletal muscle mass and significantly alters skeletal muscle miRNA expression profile in aged mice. Biochem Biophys Res Commun.

[21] Drummond MJ, Fry CS, Glynn EL, Timmerman KL, Volpi E, Rasmussen BB (2010). Aging is associated with a dysregulated human skeletal muscle microRNA-499 and-208b expression following resistance exercise. FASEB J.

[22] Drummond MJ, McCarthy JJ, Sinha M, Spratt HM, Volpi E, Esser KA, Rasmussen BB (2011). Aging and microRNA expression in human skeletal muscle: a microarray and bioinformatics analysis. Physiol Genomics.

[23] Hu Z, Klein JD, Mitch WE, Zhang L, Martinez I, Wang XH (2014). MicroRNA-29 induces cellular senescence in aging muscle through multiple signaling pathways. Aging (Albany NY).

[24] Kwon KS, Kim JY, Lee SM, Lee KP, Kim SY (2014). Genome-wide microRNA and mRNA profiling in skeletal muscle aging. FASEB J.

[25] Rivas DA, Lessard SJ, Rice NP, Lustgarten MS, So K, Goodyear LJ, Parnell LD, Fielding RA (2014). Diminished skeletal muscle microRNA expression with aging is associated with attenuated muscle plasticity and inhibition of IGF-1 signaling. FASEB J.

[26] Mercken EM, Majounie E, Ding J, Guo R, Kim J, Bernier M, Mattison J, Cookson MR, Gorospe M, de Cabo R, Abdelmohsen K (2013). Age-associated miRNA alterations in skeletal muscle from rhesus monkeys reversed by caloric restriction. Aging (Albany NY).

[27] Chio CC, Lin JW, Cheng HA, Chiu WT, Wang YH, Wang JJ, Hsing CH, Chen RM (2013). MicroRNA-210 targets antiapoptotic Bcl-2 expression and mediates hypoxia-induced apoptosis of neuroblastoma cells. Arch Toxicol.

[28] Miranda KC, Huynh T, Tay Y, Ang YS, Tam WL, Thomson AM, Lim B, Rigoutsos I (2006). A pattern-based method for the identification of MicroRNA binding sites and their corresponding heteroduplexes. Cell.

[29] Li AL, Li HY, Jin BF, Ye QN, Zhou T, Yu XD, Pan X, Man JH, He K, Yu M, Hu MR, Wang J, Yang SC (2004). A novel eIF5A complex functions as a regulator of p53 and p53-dependent apoptosis. J Biol Chem.

[30] Taylor CA, Senchyna M, Flanagan J, Joyce EM, Cliche DO, Boone AN, Culp-Stewart S, Thompson JE (2004). Role of eIF5A in TNF-alpha-mediated apoptosis of lamina cribrosa cells. Invest Ophthalmol Vis Sci.

[31] Taylor CA, Sun Z, Cliche DO, Ming H, Eshaque B, Jin S, Hopkins MT, Thai B, Thompson JE (2007). Eukaryotic translation initiation factor 5A induces apoptosis in colon cancer cells and associates with the nucleus in response to tumour necrosis factor alpha signalling. Exp Cell Res.

[32] Sun Z, Cheng Z, Taylor CA, McConkey BJ, Thompson JE (2010). Apoptosis induction by eIF5A1 involves activation of the intrinsic mitochondrial pathway. J Cell Physiol.

[33] Hilder TL, Carlson GM, Haystead TA, Krebs EG, Graves LM (2005). Caspase-3 dependent cleavage and activation of skeletal muscle phosphorylase b kinase. Mol Cell Biochem.

[34] Xiao R, Ferry AL, Dupont-Versteegden EE (2011). Cell death-resistance of differentiated myotubes is associated with enhanced anti-apoptotic mechanisms compared to myoblasts. Apoptosis.

[35] Galluzzi L, Zamzami N, de La Motte Rouge T, Lemaire C, Brenner C, Kroemer G (2007). Methods for the assessment of mitochondrial membrane per-meabilization in apoptosis. Apoptosis.

[36] Drummond MJ, McCarthy JJ, Sinha M, Spratt HM, Volpi E, Esser KA, Rasmussen BB (2011). Aging and microRNA expression in human skeletal muscle: a microarray and bioinformatics analysis. Physiol Genomics.

[37] Shang FF, Xia QJ, Liu W, Xia L, Qian BJ, You L, He M, Yang JL, Wang TH (2016). miR-434-3p and DNA hypomethylation co-regulate eIF5A1 to increase AChRs and to improve plasticity in SCT rat skeletal muscle. Sci Rep.

[38] Benne R, Brown-Luedi ML, Hershey JW (1978). Purification and characterization of protein synthesis initiation factors eIF-1, eIF-4C, eIF-4D, and eIF-5 from rabbit reticulocytes. J Biol Chem.

[39] Jao DL, Chen KY (2006). Tandem affinity purification revealed the hypusine-dependent binding of eukaryotic initiation factor 5A to the translating 80S ribosomal complex. J Cell Biochem.

[40] Zanelli CF, Maragno AL, Gregio AP, Komili S, Pandolfi JR, Mestriner CA, Lustri WR, Valentini SR (2006). eIF5A binds to translational machinery components and affects translation in yeast. Biochem Biophys Res Commun.

[41] Saini P, Eyler DE, Green R, Dever TE (2009). Hypusine-containing protein eIF5A promotes translation elongation. Nature.

[42] Li AL, Li HY, Jin BF, Ye QN, Zhou T, Yu XD, Pan X, Man JH, He K, Yu M, Hu MR, Wang J, Yang SC (2004). A novel eIF5A complex functions as a regulator of p53 and p53-dependent apoptosis. J Biol Chem.

[43] Marzetti E, Leeuwenburgh C (2006). Skeletal muscle apoptosis, sarcopenia and frailty at old age. Exp Gerontol.

[44] Dirks A, Leeuwenburgh C (2002). Apoptosis in skeletal muscle with aging. Am J Physiol Regul Integr Comp Physiol.

[45] Marzetti E, Groban L, Wohlgemuth SE, Lees HA, Lin M, Jobe H, Giovannini S, Leeuwenburgh C, Carter CS (2008). Effects of short-term GH supplementation and treadmill exercise training on physical performance and skeletal muscle apoptosis in old rats. Am J Physiol Regul Integr Comp Physiol.

[46] Marzetti E, Wohlgemuth SE, Lees HA, Chung HY, Giovannini S, Leeuwenburgh C (2008). Age-related activation of mitochondrial caspase-independent apoptotic signaling in rat gastrocnemius muscle. Mech Ageing Dev.

[47] Wohlgemuth SE, Seo AY, Marzetti E, Lees HA, Leeuwenburgh C (2010). Skeletal muscle autophagy and apoptosis during aging: effects of calorie restriction and life-long exercise. Exp Gerontol.

[48] Mohamed JS, Wilson JC, Myers MJ, Sisson KJ, Alway SE (2014). Dysregulation of SIRT-1 in aging mice increases skeletal muscle fatigue by a PARP-1-dependent mechanism. Aging (Albany NY).

[49] Ryan MJ, Jackson JR, Hao Y, Leonard SS, Alway SE (2011). Inhibition of xanthine oxidase reduces oxidative stress and improves skeletal muscle function in response to electrically stimulated isometric contractions in aged mice. Free Radic Biol Med.

[50] Ryan MJ, Jackson JR, Hao Y, Williamson CL, Dabkowski ER, Hollander JM, Alway SE (2010). Suppression of oxidative stress by resveratrol after isometric contractions in gastrocnemius muscles of aged mice. J Gerontol A Biol Sci Med Sci.

[51] Jackson JR, Ryan MJ, Alway SE (2011). Long-term supplementation with resveratrol alleviates oxidative stress but does not attenuate sarcopenia in aged mice. J Gerontol A Biol Sci Med Sci.

[52] Leeuwenburgh C, Wagner P, Holloszy JO, Sohal RS, Heinecke JW (1997). Caloric restriction attenuates dityrosine cross-linking of cardiac and skeletal muscle proteins in aging mice. Arch Biochem Biophys.

[53] Mohamed JS, Lopez MA, Cox GA, Boriek AM (2013). Ankyrin repeat domain protein 2 and inhibitor of DNA binding 3 cooperatively inhibit myoblast differentiation by physical interaction. J Biol Chem.

[54] Mohamed JS, Lopez MA, Boriek AM (2010). Mechanical stretch up-regulates microRNA-26a and induces human airway smooth muscle hypertrophy by suppressing glycogen synthase kinase-3β. J Biol Chem.

[55] Mohamed JS, Hajira A, Pardo PS, Boriek AM (2014). MicroRNA-149 inhibits PARP-2 and promotes mitochondrial biogenesis via SIRT-1/PGC-1α network in skeletal muscle. Diabetes.

[56] Vlachos IS, Zagganas K, Paraskevopoulou MD, Georgakilas G, Karagkouni D, Vergoulis T, Dalamagas T, Hatzigeorgiou AG (2015). DIANA-miRPath v3.0: deciphering microRNA function with experimental support. Nucleic Acids Res.

[57] Dweep H, Gretz N, Sticht C (2014). miRWalk database for miRNA-target interactions. Methods Mol Biol.

[58] Pardo PS, Mohamed JS, Lopez MA, Boriek AM (2011). Induction of Sirt1 by mechanical stretch of skeletal muscle through the early response factor EGR1 triggers an antioxidative response. J Biol Chem.

[59] Eedunuri VK, Rajapakshe K, Fiskus W, Geng C, Chew SA, Foley C, Shah SS, Shou J, Mohamed JS, Coarfa C, O'Malley BW, Mitsiades N (2015). miR-137 Targets p160 Steroid Receptor Coactivators SRC1, SRC2, and SRC3 and Inhibits Cell Proliferation. Mol Endocrinol.

